# Analysis of Müller glia specific genes and their histone modification using Hes1-promoter driven EGFP expressing mouse

**DOI:** 10.1038/s41598-017-03874-8

**Published:** 2017-06-15

**Authors:** Kazuko Ueno, Toshiro Iwagawa, Genki Ochiai, Hideto Koso, Hiromitsu Nakauchi, Masao Nagasaki, Yutaka Suzuki, Sumiko Watanabe

**Affiliations:** 1 Division of Molecular and Developmental Biology, Institute of Medical Science, University of Tokyo, Tokyo, Japan; 2 Division of Biomedical Information Analysis, Department of Integrative Genomics, Tohoku Medical Megabank Organization, Tohoku University, Sendai, Miyagi Japan; 3 Division of Stem Cell Therapy, Center for Stem Cell Biology and Regenerative Medicine, Institute of Medical Science, University of Tokyo, Tokyo, Japan; 4 Department of Medical Genome Sciences, Graduate School of Frontier Sciences, University of Tokyo, Chiba, Japan

## Abstract

Retinal neurons and Müller glia are generated from a common population of multipotent retinal progenitor cells. We purposed to identify Müller glia-specific molecular signatures during retinal development. Using transgenic mice carrying the *Hes1* promoter (pHes1) followed by EGFP, we purified EGFP-positive Müller glia and other EGFP-negative retinal cells from developing retinas and subjected them to RNA sequencing analysis. Gene expression pattern of EGFP-positive cell was similar to genes expressed in retinal progenitors, and they were downregulated in other cell lineages. Then, we examined the modification profiles of H3K27me3 and H3K4me3 by referring to chromatin immunoprecipitation-sequencing data of rods and other cells. Clustering of the H3K4me3 and H3K27me3 values followed by ontology analysis revealed a high incidence of transcription factors including *Hes1* in clusters with high H3K27me3 levels. *Hes1* expression level decreased dramatically, and the H3K27me3 level at the *Hes1*-locus was upregulated strongly during retinal development. Furthermore, the *Hes1* expression level was upregulated in an *Ezh2*-knockout retina. These results suggest that downregulation of Müller glia-related genes in other lineage rather than upregulation of them in Müller glia contributed Müller-specific molecular features, and a role for modified H3K27me3 in suppressing Müller glia-related genes in other retinal cell lineages to avoid unfavorable expression.

## Introduction

The vertebrate neural retina is organized into a laminar structure comprising multiple types of neurons and glial cells. Müller glia are the only glial cells derived from retinal progenitors, and their cell bodies are localized in the inner nuclear layer (INL). The major retinal cell classes in mice, including Müller glia, are generated from a common population of multipotent retinal progenitor cells that appear between embryonic day (E)11 and postnatal day (P)10 in a conserved temporal order^1^. This process is regulated by various molecular mechanisms, including transcriptional and post-transcriptional regulation.

We have been analyzing the roles of histone H3K27 methylation in retinal development^2,3^. Di and trimethylation of lysine 27 on histone H3 (H3K27me2/3) by Ezh2/Kmt6 together with polycomb repressive complex 2 acts as a gene repression mechanism^4–6^. We reported previously that the H3K27me3-specific demethylase Jmjd3 plays pivotal roles in maturing subsets of bipolar cells by demethylating *Vsx1* and *Bhlhb4* gene loci, which are essential for maturation of bipolar subsets^2^. On the other hand, the eyes of *Ezh2* retina specific-knockout mice are microphthalmic, and postnatal proliferation is diminished^3,7^. In addition, early-onset rod photoreceptors and Müller glia cells are observed in these mice. These results suggest a role for H3K27me3 in defining the timing of neurogenesis and gliogenesis, and that a more detailed analysis of the cell lineage-specific roles of H3K27me3 is necessary. In contrast, H3K4me3, which is one of most well-studied histone methylations, acts on transcription of target genes^8^, and reportedly plays a role in photoreceptor differentiation^9^.

The rod photoreceptors constitute more than 70% of all cells in the mouse retina; therefore, data obtained using the whole retina often represent the rod photoreceptors. To explore the molecular signature of rods and other retinal subsets, we examined the H3K4me3 and H3K27me3 modifications of the rod lineage and those of other cells independently using purified developing retinal subsets. Commitment to the rod lineage occurs from E12 to P7–8 in mice, and peak differentiation occurs after birth^10,11^. We purified rod and photoreceptor precursors using specific expression of the cell surface marker Cd73^12^ from postnatal developing retinas, and we also purified the Cd73-negative population, which consists of interneurons, Müller glia, and RGCs. RNA sequencing (RNA-seq) and chromatin immunoprecipitation sequencing (ChIP-seq) for H3K4me3 and H3K27me3 were performed^13^.

In this study, we focused on Müller glia-specific molecular events using the retinas from *Hes1* promoter-enhanced green fluorescent protein (EGFP) transgenic mice.

## Methods

### Mice

All animal experiments were approved by the Animal Care Committee of the Institute of Medical Science, University of Tokyo and conducted in accordance with the ARVO (Association for Research in Vision and Ophthalmology) statement for the use of animals in ophthalmic and vision research. ICR mice were obtained from Japan SLC Co. The transgenic mice of 2.5-kb promoter of *Hes1* followed by destabilized *EGFP* (pHes1-EGFP mouse)^14^ was kindly provided by Dr. Ryoichiro Kageyama.

### RNA sequencing (RNA-seq) and quantification

Total RNA was extracted from stored purified cell pellets using RNeasy Plus Micro Kit (Qiagen), and RNA quality was confirmed using a 2100 Bioanalyzer (Agilent Technologies). RNA-seq was done as previously described^13,15^. The original data set were deposited in GEO (GSE86199). The sequence reads were aligned to the mouse reference genome assembly (NCBI37/mm9) using TopHat (v2.0.8b) with the option (–coverage-search –max-multihits 50)^16^. Then, fragments per kilobase of exon per million mapped fragments (FPKM) was calculated from the aligned reads by using CuffLinks (v2.1.1) with the default option^16^ as previously described^13^. By using CuffLinks, 23,021 genes expression values (FPKM) were estimated, and small RNAs such as miRNAs were excluded, and remaining 19,954 genes were subjected to following analyses. K-means clustering was done using GeneSpring software (v. 12.6.1; Agilent Technologies). Flow of data analysis is shown in Supplemental Fig. 1. To verify H3K27me3 levels of genes in the clusters in pHes1_EPC and pHes1_ENC, three representative genes in each cluster were selected as follows. For each gene, the sum total of H3K27me3 level of Cd73 (P2N, P2P, P5N, P5P) was calculated, and top 18 genes from the biggest value were selected. Then, a square value of difference between P2N and P2P, and P5N and P5P were calculated. For C1, C2, C4, and C5 top 3 genes with from smallest difference were selected, and for C3, C6, and C7, top 3 genes with largest difference were selected as representative genes.

### Immunostaining, Retinal explant culture, RT-qPCR, ChIP-quantitative polymerase chain reaction (ChIP-qPCR), and data mining of ChIP-sequencing results

Immunostaining of sections was done as described previously^17^. Primary antibodies, anti-EGFP (Chicken polyclonal, Abcam ab13970202) and anti-Glutamine synthetase antibody (Mouse monoclonal, Chemicon MAB30219), were visualized by using Alexa Fluor 488 or 594 dyes conjugated second antibodies (Molecular Probes), respectively. Samples were mounted in PBS containing 50% glicerol and analyzed by using a Zeiss Axio Vision 4.6 microscope. Retinal explant culture was performed at 34 °C on a chamber filter (Millicell) as previously described^17^. ChIP-qPCR was done as previously described^2^. For RT-qPCR, total RNA was purified from homogenized tissues using Sepasol RNA I Super G (nacalai tesque), and cDNA was synthesized using ReverTra Ace pPCR RT Master Mix (TOYOBO). Quantitative PCR (qPCR) was performed using the SYBR Green-based method, using the Roche Light Cycler 96 (Roche Diagnostics). *Glyceraldehyde 3-phosphate dehydrogenase* (*Gapdh*) was used as an internal control. The binding of Ezh2 was examined using anti-Ezh2 antibody (Active Motif, 39875) and mouse IgG (BioLegend, 401302). The histone modification value of H3K4me3 and H3K27me3 were calculated as follows. For test samples and input samples, the 1050 bp-window tags (50 bp sliding) were counted, and number of tags at each position was divided by the number of total tags. The values of the test samples were subtracted by that of input samples at each genome position. The values were converted into log2 scale, and the averaged values in TSS +/−5 kb (Refseq genes except for small RNAs) were subjected to quantile normalization.

### Statistical analysis

**Figure 1:**
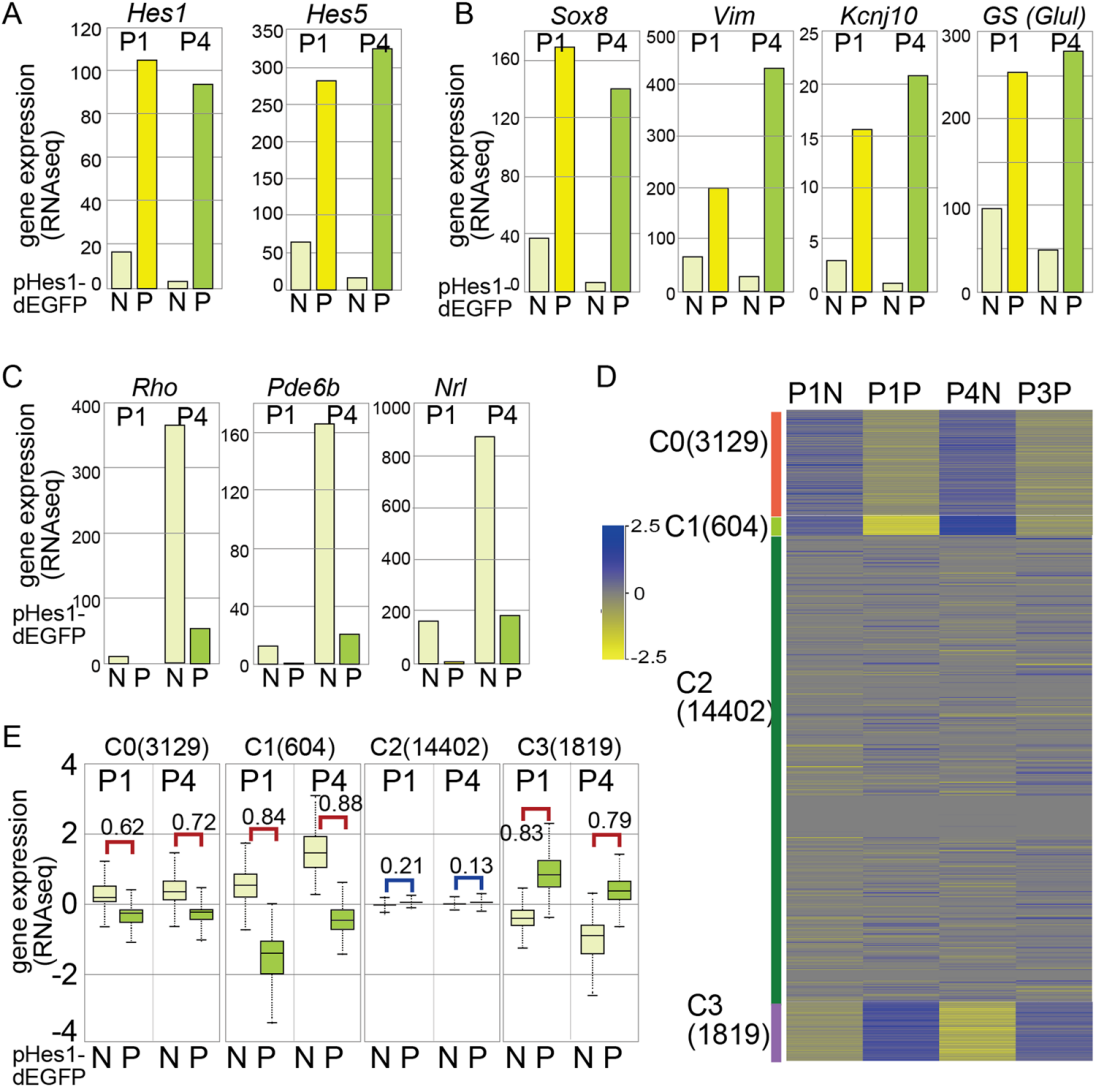
Fractionation of retinal cells by EGFP expression from pHes1-EGFP transgenic mouse retina. (**A**,**B**,**C**) RNA-seq using purified EGFP positive (P) and negative (N) cells of retina from *pHes1*-EGFP mouse at P1 and P4 was done. RNA-seq values of genes related to Müller glia (**A**,**B**) or photoreceptor (**C**) are shown. (**D**,**E**) Unsupervised K-means clustering of all genes on the RNA-seq to generate 4 clusters was done (**D**), and the distribution of gene expression of the clusters 0, 1, 2, and 3 in pHes1-EGFP positive (P) and negative (N) populations are shown (**E**). Numbers in parentheses are gene number in each cluster. Statistical analysis was done by student’s t test, and effect size of the results was examined by Pearson’s correlation coefficient (r) and indicated in the figure. N, negative; P, positive.

**Figure 2:**
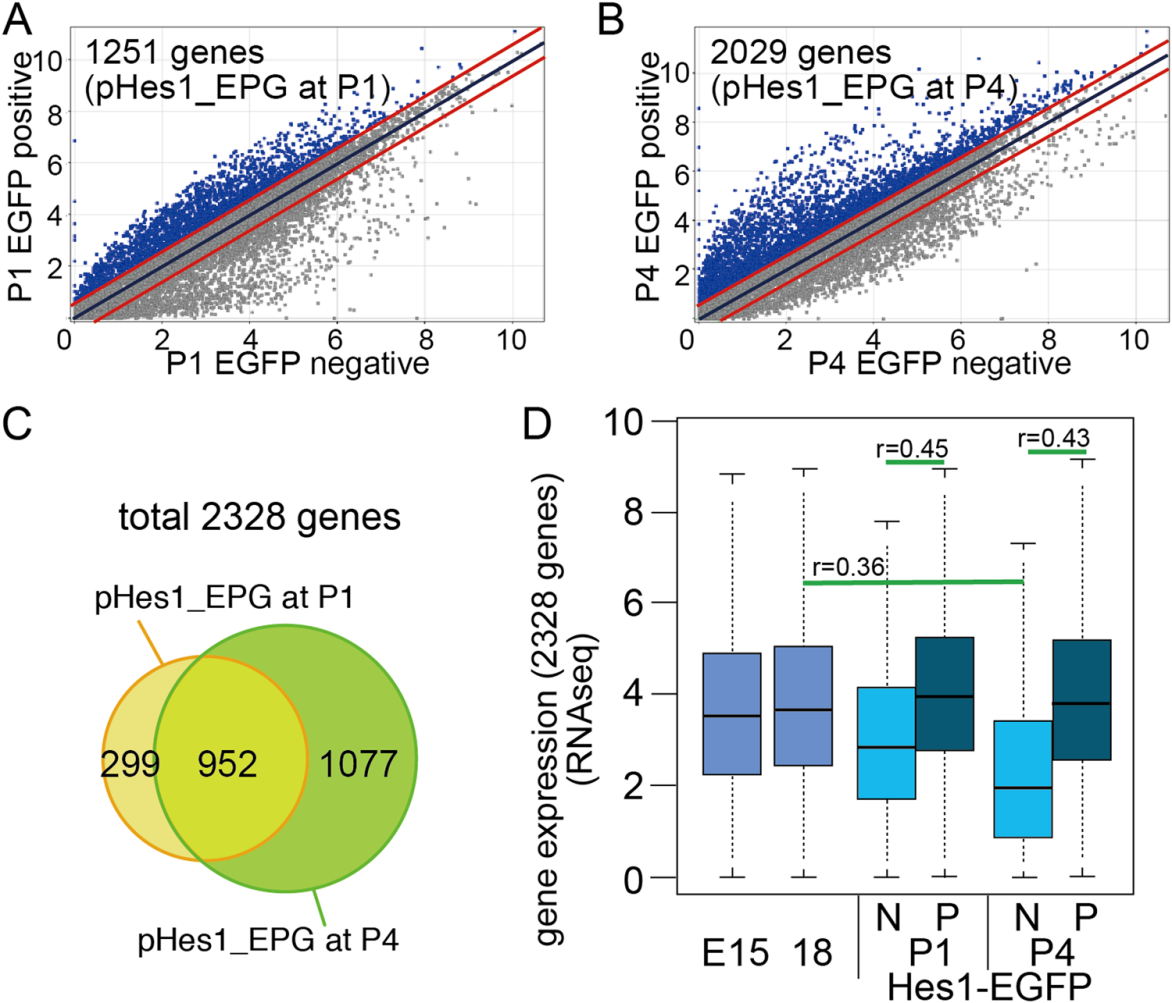
Identification of pHes1-EGFP positive cell specific genes. (**A**,**B**) Global gene expression levels of EGFP negative (horizontal axis) and EGFP positive (vertical axis) pattern of RNA-seq derived from P1 (**A**) and P4 (**B**) *pHes1*-EGFP mouse retina. Diagonal lines indicate median (middle line) and +/− 2 fold difference values (upper and lower lines). Genes predominantly expressed in either fraction was designated as pHes1-EPG and pHes1-ENG only if expressed at >2 fold than opponent fraction. (**C**) Venn diagram showing overlapping of genes of pHes1-EPG at P1 vs. pHes1-EPG at P4. (**D**) Average and distribution of gene expression levels of 2,328 genes, which expressed in higher in pHes1-EPG at P1 and/or P4. RNA-seq data of whole retina at E15 and E18^13^, and pHes1-EPC and -ENC are shown. N, negative; P, positive. The p values were calculated by student’s T test, and effect size of the results was examined by Pearson’s correlation coefficient (r).

**Figure 3:**
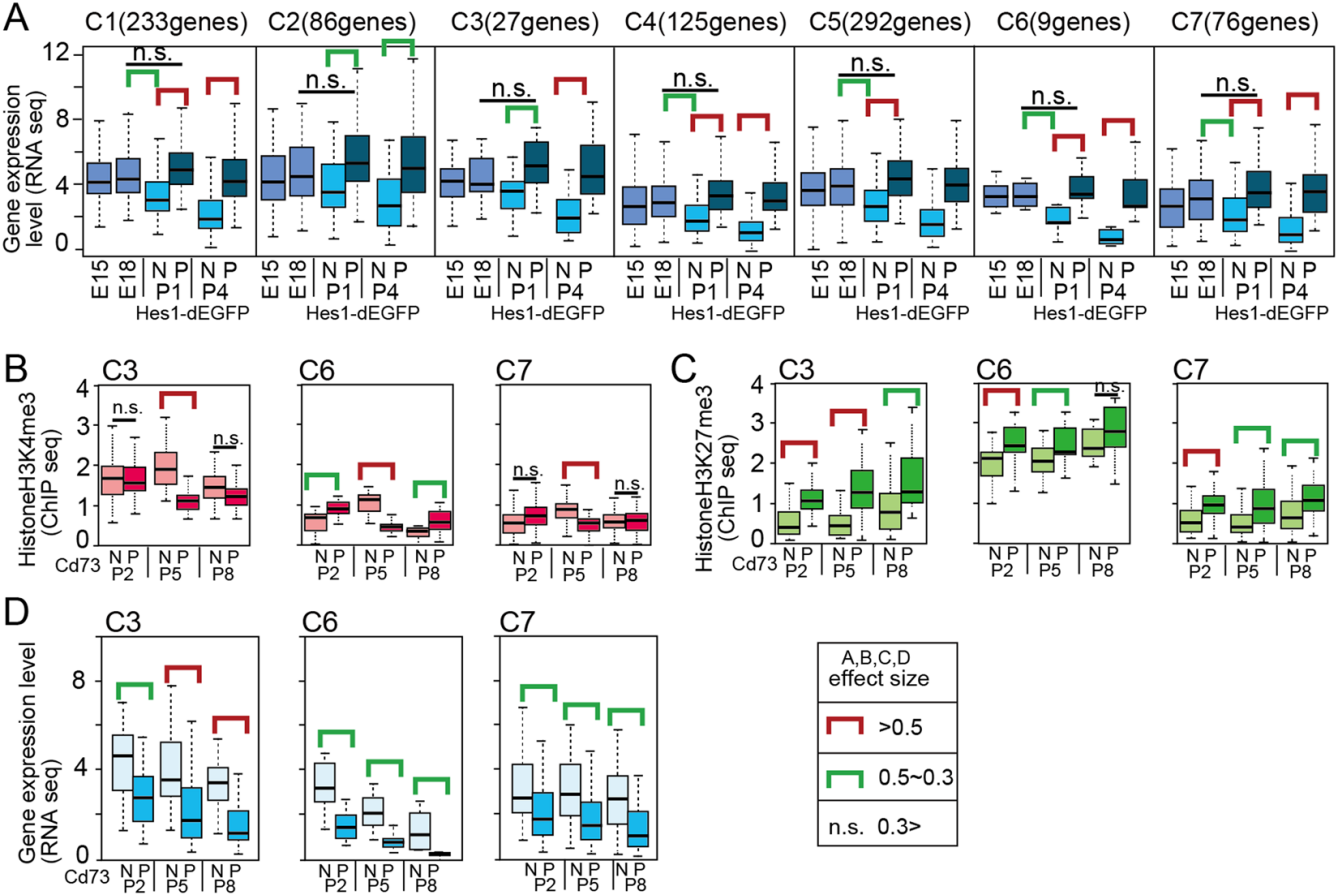
Characterization of genes commonly upregulated in P1 and P4 pHes1 EGFP positive cell populations. (**A**) Genes that expressed higher levels in pHes1-EPC than in pHes1-ENC at both P1 and P4 were sub-clustered to C1 to C7 by using their values of H3K4me3 and H3K27me3 ChIP-seq of Cd73_P and Cd73_N retinal fractions^13^. Average and distribution of gene expression levels (RNA-seq) are shown by box plot. RNA-seq data obtained from whole retina at E15 and E18^13^ are also shown. (**B**–**D**) Distribution of H3K4me3 (ChIP-seq, **B**), H3K27me3 (ChIP-seq, **C**), and transcripts level (RNA-seq, **D**) of genes in each sub-cluster are shown. ChIP-seq and RNA-seq were performed by using Cd73_P and Cd73_N retinal sub-fractions at P2, P5, and P8^13^. Green and red bars indicate statistical significant. p values were calculated by student’s T test, and effect size of the results was examined by Pearson’s correlation coefficient (r). r; Red bar >0.5, 0.5> green bar >0.3, n.s. <0.3.

For Figs 1, 2 and 3, the p values were calculated by student’s T test, and effect size of the results was examined by Pearson’s correlation coefficient (r).

### Primers sequences for qPCR

*Hes1* 5′-cggtctacaccagcaacagt-3′, 5′-aagagagaggtgggctaggg-3′

*Ezh1* 5′-catgacccagaacttttgtgaa-3′, 5′-gacaaccaggaaagcgattc-3′

Primers not listed had been published previously in ref.^2^.

### Primer sequences for ChIP-qPCR

*Epha3* 5′-aaaacggaatggctcccaag-3′, 5′-ttgctggtggggtaggaaag-3′

*Chst11* 5′-tgtcacctaacctgcttctcg-3′, 5′-tgcgtgaacgtttccatgac-3′

*Al504432* 5′-aagccagtgagagtcagtgtac-3′, 5′-tctgcttttccagtgctgtg-3′

*Grid2* 5′-aaaaaccgtcaaggcaggtg-3′, 5′-aaaaccgcgctctcaattcc-3′

*Irx5* 5′-acacaaggcgtcagaatcctc-3′, 5′-gggctctgcttttggctttg-3′

*Tubb6* 5′-actaacagtaccgtggtgcag-3′, 5′-ttggtcctgacagctggaac-3′

*Cdc42ep1* 5′-aggtcatctaaggagcagtgg-3′, 5′-tcgggaatgttcgctctgtc-3′

*Ugt8a* 5′-tctggagcggagactttgatg-3′, 5′-tcaagccgtctctcaaacctc-3′

*Il1rap* 5′-tcttcagtggagcacaacgg-3′, 5′-aacgtgcaaggaagggctag-3′

*Irx3* 5′-caactttaagccgccttagctc-3′, 5′-gcttttgctcacctttctgc-3′

*Cyp26a1* 5′-aacccaggactccaaattccc-3′, 5′-tctttccttgtcctaggtgtgc-3′

*Sox3* 5′-aagctctcatcacgtcactcg-3′, 5′-tgatgagtcctggccaatcaac-3′

*Fxyd7* 5′-tttcggacaccccttgctg-3′, 5′-ttttcgcacaagaggcactg-3′

*Fxyd1* 5′-atcaaaggctggcagaacac-3′, 5′-tgctaggtttcctgggaatcag-3′

*Cyr61* 5′-gccaaccaacattcctgagatg-3′, 5′-agcccgccctttataatgcc-3′

*Itgb8* 5′-acctgtggaagaagctagcac-3′, 5′-tcacagggtgatgctgctttag-3′

*Ccnd1* 5′-acattcttccttggcttgcg-3′, 5′-ggggcttctttccctaagagg-3′

*Irs1* 5′-ccggaatgtagagcgagcag-3′, 5′-agaggagcaaaacacgtgac-3′

*Oaf* 5′-aagccagcctagaccacaaag-3′, 5′-aacagaaaccaccaggacctg-3′

*Etv5* 5′-gacctctgagggcgttgag-3′, 5′-tcttcaggcaaatccagctg-3′

*Hey2* 5′-cctctgccaacgcctctc-3′, 5′-acggtgtggctttctattgg-3′

### Construction of plasmids encoding sh-RNA against Ezh1

For the construction of the shRNA vectors, a target sequence was selected using Gene Link RNAi Explorer (http://www.genelink.com). BLAST analysis showed no homology between each target sequence and any other sequence in the mouse genome database. Double-stranded DNA for the target sequences was constructed as previously^18^ using pU6 shRNA expression vector^19^. Target sequences are as follows; sh-Ezh1_1: 5′-gaaagataacaattctacaca-3′, sh-Ezh1_2: 5′-gaattcatttctgaatattgt-3′.

### Flow cytometry and cell sorting

Retinas were isolated from enucleated eyes, and incubated with 0.25% Trypsin in PBS (500 μl) for 15 minutes at 37 °C. Samples were treated with DNaseI and stained with propidium iodide. EGFP positive and negative fractions were purified by using MoFlo cell sorter (Beckman Coulter). Purified cells were stored at −80 °C as cell pellet until use for RNA-seq.

### Ingenuity pathway analysis

Categorized genes were imported into the Ingenuity Pathway Analysis (IPA; QIAGEN bioinformatics; https://www.qiagenbioinformatics.com/products/ingenuity-pathway-analysis/). In IPA each gene was assigned molecular type or biological function such as transcription regulator, cytokine, growth factor, and so on. The basis of the IPA program consists of the Ingenuity Pathway Knowledge Base (IPKB) which is derived from known functions and interactions of genes published in the literature.

## Results

### Comprehensive analysis of the gene expression patterns of Hes1 promoter (pHes1)-driven EGFP-positive and -negative populations

Hes1 is a well-known transcription factor expressed by Müller glia in the retina^20^. Transgenic mice harboring the 2.5-kb *Hes1* promoter followed by dEGFP (pHes1-EGFP) expressed EGFP in almost all Müller glia in the postnatal retina^14^. We confirmed that specific expression of EGFP continued in Müller glia in the adult retina (Supplemental Fig. 2A and B).

To analyze the molecular signatures of the Müller glia, we purified *pHes1-*driven EGFP-positive cells (pHes1_EPCs) and -negative cells (pHes1_ENCs) at P1 and P4 using a cell sorter, and the purified cells were subjected to RNA-seq analysis. The RNA-seq data showed approximately 10- and 30-fold greater enrichment of *Hes1* transcripts in the pHes1_EPC population than in the pHes1_ENC population at P1 and P4, respectively (Fig. 1A). Similar results were obtained for *Hes5* expression (Fig. 1A). We then analyzed the expression of known genes expressed specifically in retinal sub-populations. Müller glia-specific genes^21–24^ were expressed dominantly in pHes1_EPCs (Fig. 1B). Expression of the rod photoreceptor-specific genes was low in both fractions at P1 because rod differentiation occurs mainly postnatally and was expressed in pHes1_ENCs at P4 (Fig. 1C).

To verify the RNA-seq data, we performed unsupervised K-means clustering and all genes in the RNA-seq to generate four clusters, which provided good resolution (Table S1). The gene expression levels (log transformed) across the four samples are shown as a heat map (Fig. 1D). Genes with higher median expression levels in pHes1-ENCs than in pHes1-EPCs were clustered as 0 and 1 (Fig. 1E). We subjected genes in clusters 0, 1, and 3 to an Ingenuity Pathway Analysis (Table S1). The genes categorized as transcription regulators are shown in Table S1. Transcription factors known to be involved in differentiation of Müller glia were found in C3 (Table S1, C3_TF), and those in the photoreceptor cell lineage were found in C1 (Table S1, C1_TF). These results confirm that *pHes1*-driven EGFP-dependent separation of retinal cells helped purify the Müller glia population.

### Identification of Müller glia-specific genes

We considered *pHes1*-positive population-specific genes only if they had more than 2-fold higher absolute expression levels in pHes1_EPCs than in pHes1_ENCs. In total, 1,251 and 2,029 genes at P1 and P4, respectively, were listed as pHes1_EPC-specific genes (pHes1_EPGs) (Fig. 2A,B, genes above upper red line). Among them, 952 genes were upregulated in common in the pHes1_EPC population at P1 and P4 (Fig. 2C), therefore total 2,328 genes were identified as genes expressed in Hes1-EPC.

Interestingly, when we examined the expression levels of the 2,328 genes in retinal progenitors using our RNA-seq data^13^, the average levels of the genes in progenitors at E15 and E18 and in Hes1-positive cells were similar, and it was down regulated at Hes1-negative population at P4 (Fig. 2D). We then characterized the histone modifications at Müller glia-specific gene loci. We had previously performed a ChIP-seq analysis of H3K4me3 and H3K27me3 using Cd73-positive rod photoreceptors (Cd73_PCs) and other Cd73-negative cells (Cd73_NCs), including Müller glia in the developing mouse retina^13^. As photoreceptors constitute more than 70% of the total retinal population, signals from retinal sub-fractions other than rods should be stronger in the Cd73_NC population compared with that in the entire retina, although the values are the sum of multiple retinal sub-populations. Among the 952 genes (Fig. 2C), H3K4me3 and H3K27me3 values of 848 genes were detected in the ChIP-seq data^13^. We clustered the 848 genes by hierarchical clustering using the H3K4me3 and H3K27me3 values in Cd73_PCs and Cd73_NCs and obtained seven clusters, which were designated C1–C7 (Fig. 3A and Table S2). As expected, average RNA-seq values in the clusters were higher in pHes1_EPCs than in pHes1_ENCs (Fig. 3A). We had previously performed an RNA-seq analysis of retinal progenitors using whole retinas at E15 and E18^13^. Again, RNA-seq values of E18 retinas and pHes1_EPCs did not differ significantly; however, those of E18 retinas and pHes1_ENCs at P1 differed significantly in C1, 4, 5, 6, and 7 (Fig. 3A). These results suggested that pHes1_EPCs exhibited a gene expression pattern similar to that of retinal progenitor cells, and that the expression levels of genes specific to pHes1_ENCs decreased compared to genes in other cell lineages. We performed clustering using the H3K4me3 and H3K27me3 values. The H3K4me3 values were generally low and were particularly low in Cd73_PCs (Fig. 3B and Supplemental Fig. 3A). These results are consistent with higher expression of genes in the Cd73_NC population than the Cd73_PC population (Fig. 3D and Supplemental Fig. 3C), as Müller glia are included in the Cd73_NC population.

H3K27me3 levels were very low with no difference between Cd73_NCs and Cd73_PCs in the clusters except for C3, C6, and C7, which showed significantly higher levels in Cd73_PCs than in Cd73_NCs (Fig. 3C and Supplemental Fig. 3B). The genes in C3 and C6 showed very low expression levels in the Cd73_PC population, which was consistent with higher levels of H3K27me3 in Cd73_PCs (Fig. 3C,D and Supplemental Fig. 3B,C).

### Differential H3K27me3 levels of pHes1_EPC-specific genes loci in pHes1-EPC and pHes1-ENC

**Figure 4:**
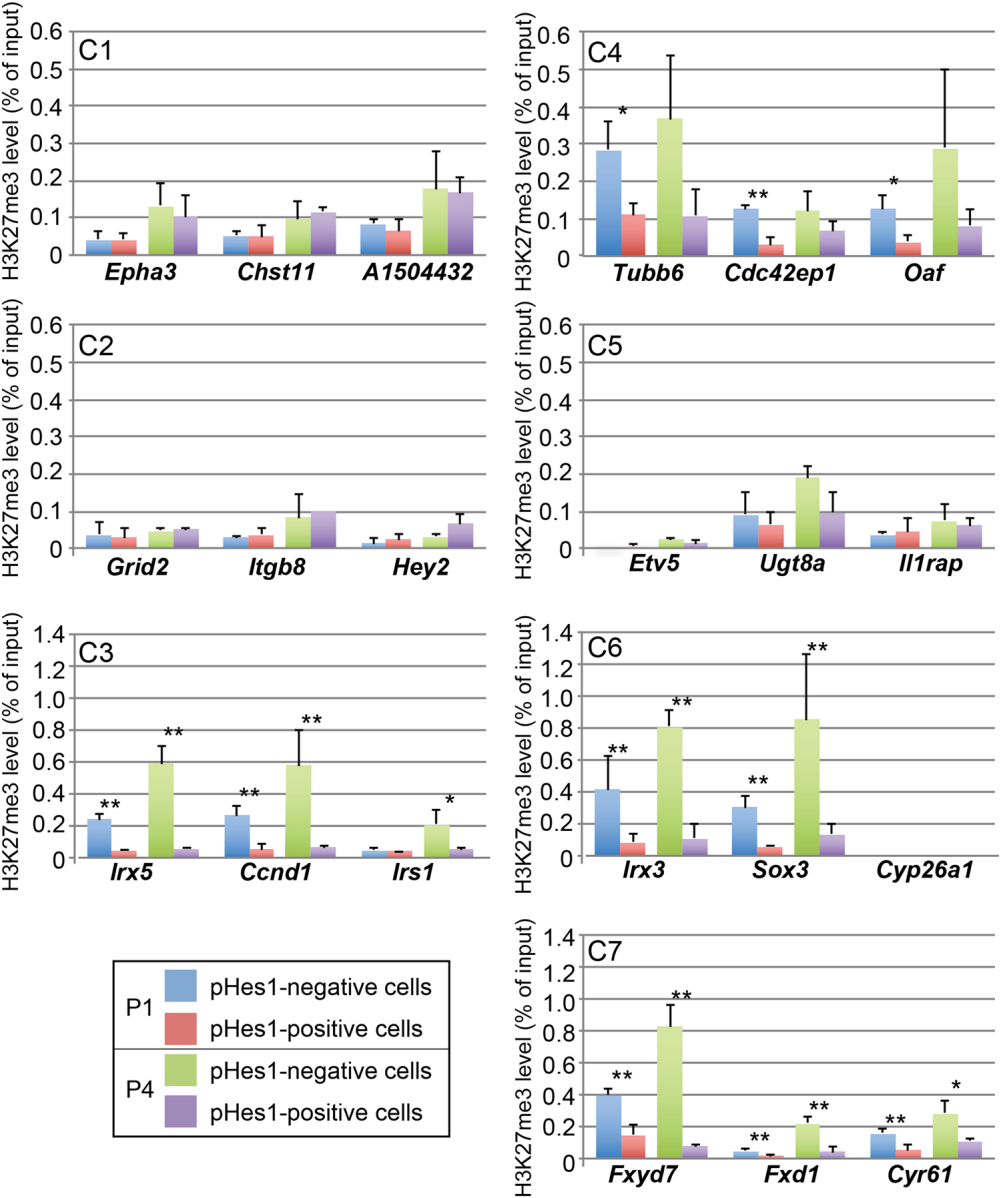
H3K27me3 levels of pHes1_EPC-specific genes loci in pHes1-EPC and pHes1-ENC. H3K27me3 levels of representative genes of each cluster in pHes1-EPC and pHes1-ENC at P1 and P4. ChIP using anti-H3K27me3 antibody was done using extracts of purified pHes1-EPC and pHes1-ENC from mouse retina at P1 and P4. qPCR was done using representative genes in each cluster. The experiments were done three times, and values are average of three samples with standard deviation. p value; **<0.01, *<0.05, or n.s. >0.05 was calculated by Student’s t-test.

We then examined H3K27me3 levels of representative genes of each cluster in pHes1-EPC and pHes1-ENC at P1 and P4. The genes examined were selected as described in Methods section, H3K27me3 modification levels of the gene loci in pHes1-EPC and pHes1-ENC at P1 and P4 were examined by ChIP-qPCR. As expected the genes in C3, C6, and C7 showed significantly different levels of H3K27me3 levels (Fig. 4). The gene loci of C1, C2 C5 showed no difference between two cell groups but the genes in C4 showed significant difference in some cases (Fig. 4).

### Transcription factors are enriched in clusters C3 and C6

**Figure 5:**
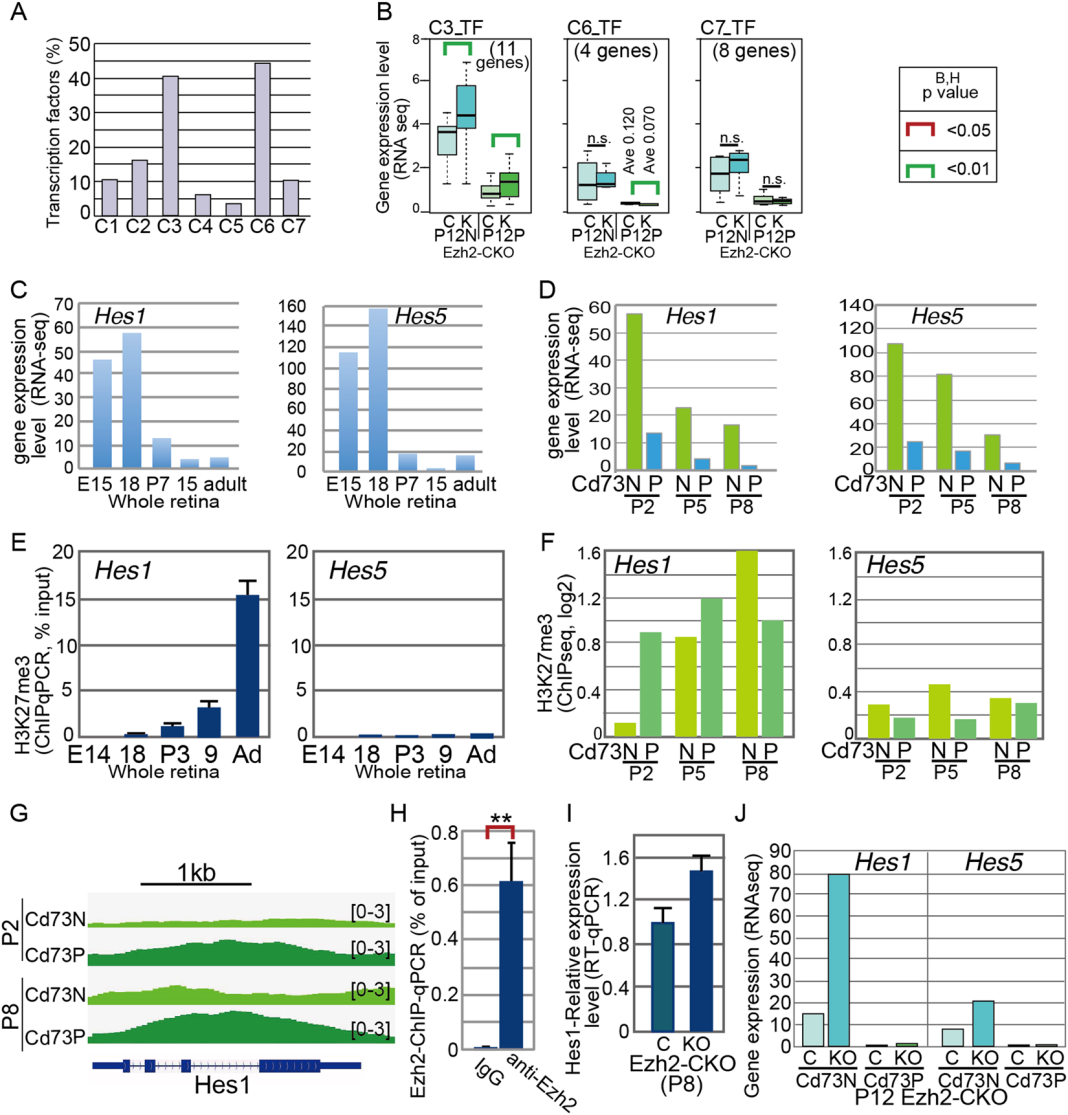
Molecular signature of Hes1 and Hes5 genes. (**A**) Percentage of genes categorized to transcription factors by IPA in total number of genes in the clusters is shown. (**B**) Box plots of average levels of transcription factors in each cluster of RNA-seq data of Ezh2-CKO retina^3^. Retinas from (**C**), control mouse; K, Ezh2-CKO mouse were^13^ used. P12N, Cd73 negative population at P12; P12P, Cd73 positive population at P12 retinas. (**C**) Expression of *Hes1* and *Hes5* transcripts during mouse retinal development examined by RNA-seq^13^ are shown. (**D**) Expression levels of *Hes1* and *Hes5* transcripts examined by RNA-seq using Cd73_P and Cd73_N cell fractions in indicated developmental stages. (**E**) H3K27me3 levels at 5′ genomic region of *Hes1* and *Hes5* loci examined by ChIP-qPCR using whole retina at indicated developmental stages using primers for *Hes1* and *Hes5* are shown. (**F**) H3K27me3 levels of *Hes1* and *Hes5* loci examined by ChIP-seq using Cd73_P and Cd73_N retinal cells. (**G**) Genome browser snapshots showing the *Hes1* region of log2 ratio enrichment for H3K27me3. (**H**) ChIP-qPCR of anti-Ezh2 antibody of retinal cell extract from control mice. (**I**) The expression level of Hes1 in retina of Ezh2-CKO^3^ at P8 examined by RT-qPCR. (**J**) The expression level of Hes1 and Hes5 in Cd73 positive and negative population in retina of Ezh2-CKO at P12 examined by RNA-seq. E, embryonic day; P, postnatal day; N, negative; P, positive. H; The experiments were done three times, and values are average of three samples with standard deviation. p value; **<0.01 was calculated by Student’s t-test.

We subjected the genes in each cluster to an ontology analysis to examine the gene classification (Table S3). Interestingly, we found a high incidence of genes categorized as transcription regulators in the C3 and C6 clusters (Table S3 and Fig. 5A). The high degree of modification of H3K27me3 in the gene loci of the C3 and C6 clusters in Cd73_PCs (Fig. 3C) suggested that the lower expression levels of these genes in rods were regulated by modifying H3K27me3. Ezh2 is a major H3K27 metnyltransferase in the retina^3^, and expression levels of transcriptional regulators in each cluster of Cd73_P and Cd73_N retinal cells at P12 in retina-specific conditional knockout (CKO) *Ezh2* mice showed that C3 genes in Cd73_PCs and Cd73_NCs were upregulated at P12 in the *Ezh2*-CKO retina^3^ (Fig. 5B and Supplemental Fig. 3D). These results suggest that the expression levels of these genes in C3 may be at least partly regulated by modifying H3K27me3.

### Hes1, but not Hes5, is suppressed by modifying H3K27me3 during the late phase of retinal development

Hes1 and Hes5 are critical transcription repressors for Müller glia differentiation^25,26^ and belonged to the C3 cluster category (Fig. 3A). The *Hes1* and *Hes5* transcripts were expressed strongly in the embryonic retina, but they were expressed only weakly in the mature retina (Fig. 5C). As expected, *Hes1* and *Hes5* were expressed in the Cd73_N population, predominantly at P2, P5, and P8, and expression levels decreased gradually in both fractions (Fig. 5D). ChIP-qPCR using whole retinal samples showed that the H3K27me3 level increased at the *Hes1* locus as retinal development proceeded (Fig. 5E). In contrast, the H3K27me3 level at the *Hes5* locus was constantly very low at all developmental stages examined (Fig. 5E). The H3K27me3-ChIP-seq data for the *Hes1* locus in Cd73_PCs and Cd73_NCs showed a strikingly higher modification level in Cd73_PCs at P2; the levels were comparable at P5, but higher in Cd73_NCs than in Cd73_PCs at P8 (Fig. 5F). The levels of H3K27me3 in Cd73_PCs at P2, P5, and P8 were similar, and we surmise that the modification was once upregulated at P2 to shut off the expression of *Hes1* when the cells started to differentiate, then kept at least until P8. The *Hes1* level was not upregulated since positive transcription regulators for *Hes1* may disappeared in the differentiated cell population. On the other hand, in Cd73_NCs, H3K27me3 level was upregulated during retinal development to actively suppress *Hes1* expression. In contrast, again, the H3K27me3 level at the *Hes5* locus was consistently very low in both the Cd73_P and Cd73_N populations (Fig. 5F). The genome browser pattern confirmed an H3K27me3 modification of the *Hes1* gene at P2 in Cd73_PCs but not in Cd73_NCs (Fig. 5G). ChIP-qPCR by using anti-Ezh2 antibody suggested Ezh2 was in *Hes1* locus (Fig. 5H). Finally, we examined *Ezh2*-CKO retinas^3^, and the *Hes1* transcript level was increased at P8 in the *Ezh2*-CKO retina (Fig. 5I). Later stage RNA-seq data showed that *Hes1* expression was increased in the Cd73_N fraction but not in the Cd73_P fraction (Fig. 5J). The increment of *Hes1* expression indicates that *Hes1* expression was released from the suppression by H3K27me3 modification. We surmised that the *Hes1* transcript was not enhanced in the Cd73_P population because of the lack of positive transcription regulators for *Hes1* in Cd73_PCs. H3K27me3 levels in Cd73_PCs were similar at P2, P5, and P8, suggesting that once the *Hes1* locus was modified with H3K27me3, the modification was kept during development. Weak upregulated expression was observed for *Hes5*; however, this effect may have been secondary to *Hes1* upregulation. *Ezh2* homologue *Ezh1* is also expressed in retina. We next asked roles of Ezh1 for regulation of *Hes1* level. Plasmids encoding sh-RNA against *Ezh1* were constructed (sh-Ezh1_1, sh-Ezh2_2), and the plasmids were transfected into NIH3T3 cells to examine the efficiency of sh-Ezh1s. After 48 hours, cells were harvested, and expression of *Ezh1* and *Ezh2* were examined by RT-qPCR (Supplemental Fig. 4A). Both sh-Ezh1_1 and sh-Ezh1_2 suppressed *Ezh1* but not *Ezh2*. Then the plasmids were transfected to retinal explants prepared from mouse embryos at E18. After 9 days of culture, the explants were harvested, and mRNA levels were examined by RT-qPCR (Supplemental Fig. 4B). The expression levels of neither *Hes1* nor *Hes5* were upregulated by the sh-Ezh1_1 or sh-Ezh1_2. *Hes1* expression was rather suppressed by both plasmids (Supplemental Fig. 4B).

### Genes have higher expression levels in the P4- but not in the P1-Hes1-EGFP population

**Figure 6:**
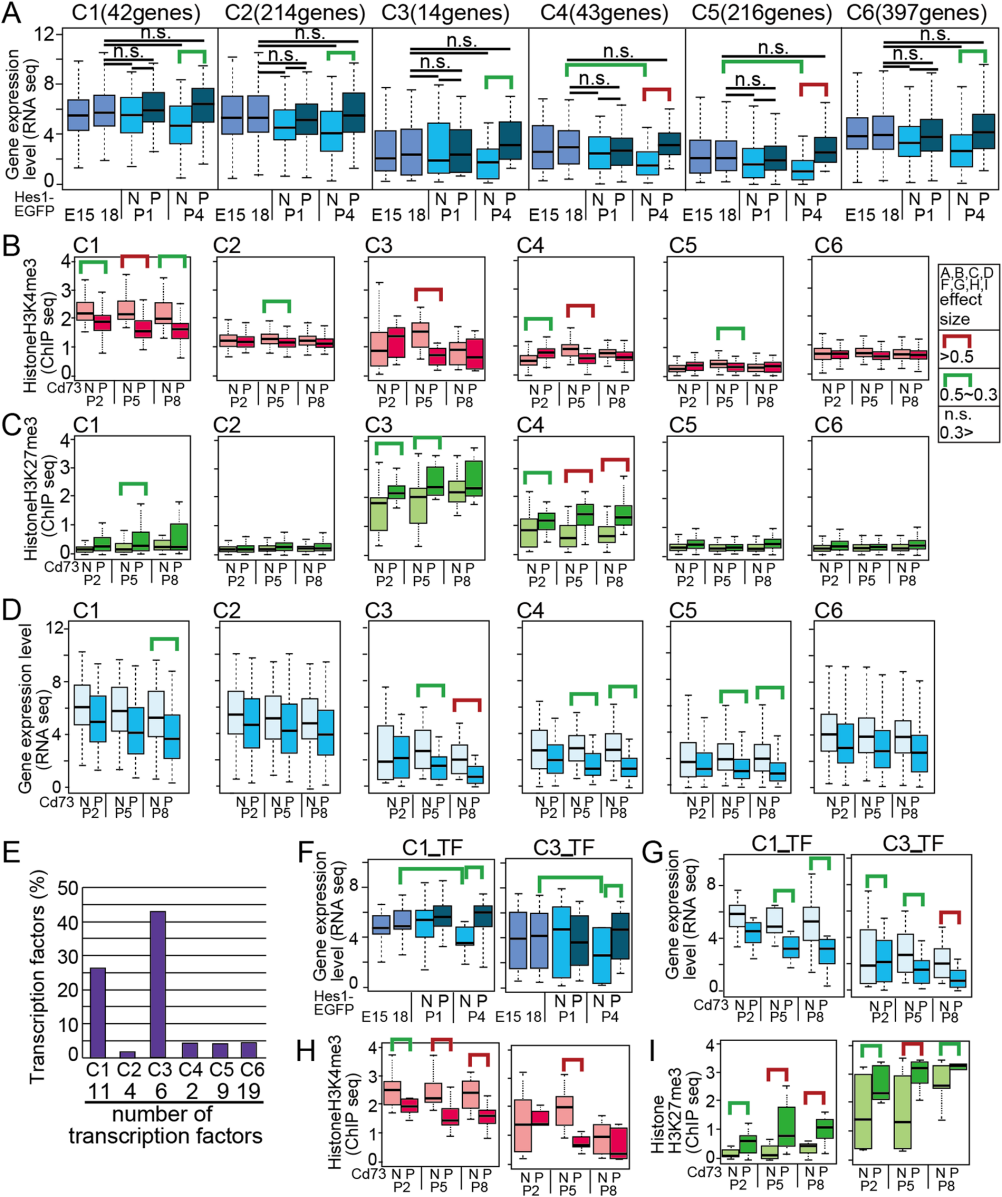
Characterization of genes upregulated in P4 but not in P1 in pHes- EGFP positive cell populations. Genes of pHes1-EPG at P4 but not included in pHes1-EPG at P1 were sub-clustered to C1 to C7 by using their values of H3K4me3 and H3K27me3 ChIP-seq of Cd73_P and Cd73_N fractions^13^, and distribution of gene expression levels (RNA-seq) in each cluster are shown by box plot (**A**). (**B**–**D**) Box plots of distribution of H3K4me3 (ChIP-seq, **B**), H3K27me3 (ChIP-seq, **C**), and transcripts level (RNA-seq, **D**) of genes in each sub-cluster are shown. ChIP-seqs and RNA-seq were performed by using Cd73_P and Cd73_N retinal sub-fractions at P2, P5, and P8^13^. (**E**) Percentage of genes categorized to transcription factors by IPA in total number of genes in the clusters is shown. (**F**–**I**) Distribution of gene expression levels in pHes1-EPC or pHes1-ENC (**F**), or in Cd73_P and Cd73_N (**G**). Levels of H3K4me3 (**H**) and H3K27me3 (**I**) of Cd73_P and Cd73_N fractions of the transcription factors in each cluster are shown.

As shown in Fig. 2C, 1,077 genes exhibited higher expression levels in pHes1_EPCs than in pHes1_ENCs at P4, but not at P1. We examined H3K4me3 and H3K27me3 levels of the gene loci and found 926 of the 1,077 genes in the Cd73_P and Cd73_N ChIP-seq data^13^. We clustered the 926 genes using the H3K4me3 and H3K27me3 values and obtained six clusters by hierarchical clustering analysis (Table S4). The mean RNA-seq values in each cluster showed higher expression levels in pHes1_EPCs than in pHes1_ENCs at P4 but not at P1 in all clusters, as expected (Fig. 6A). A comparison of the RNA-seq data for the entire retina at E15 with that at E18 showed that gene expression levels in pHes1-EPCs at P4 were not higher than at E18 in all clusters, but levels in pHes1-ENCs at P4 of clusters 4 and 5 were lower than the levels at E18, suggesting that the genes in the pHes1-EN population were downregulated at later stages of development rather than being upregulated, as in the pHes1-EP population. The H3K4me3 level in the C1 cluster was lower in the Cd73_P fraction than in the Cd73_N fraction, but no differences were detected between other clusters in the Cd73_P and Cd73_N populations with some exceptions (Fig. 6B). Expression levels were relatively broadly distributed but tended to be higher in the Cd73_N population than the Cd73_P population (Fig. 6D). Levels of H3K27me3 were higher in the C1, C3, and C4 clusters of the Cd73_P population, whereas the levels were very low in the C2, C5, and C6 clusters of the Cd73_P and Cd73_N populations (Fig. 6C). We then subjected the genes in each cluster to an ontology analysis and detected a high frequency of transcription factors in the C1 and C3 clusters (Table S5 and Fig. 6E), suggesting strict regulation of transcription factors by histone modification. In fact, we observed downregulation of expression levels of the transcription factors in C1 and C3 populations in pHes1-ENC in compare with that of the whole retina at E18 (Fig. 6F). Furthermore, analyses of expression levels and histone H3K4/27me3 of these transcription factors showed that their expression and histone modification levels were clearly different between Cd73_P and Cd73_N populations (Fig. 6G–I).

### Comparison of gene expression between pHes1- and pHes5-labelled Müller glia cells

**Figure 7:**
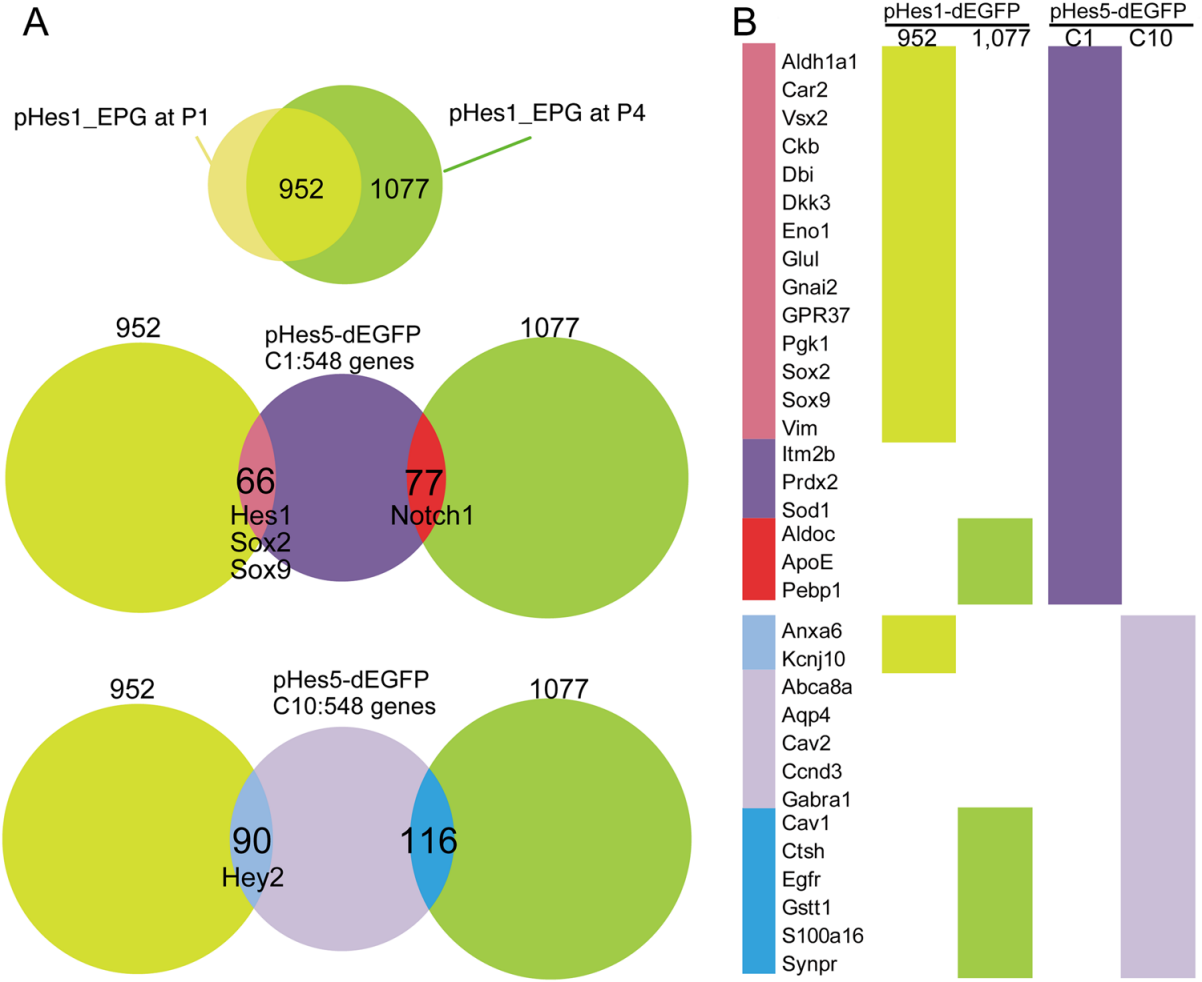
Comparison of gene expression patterns of pHes1-EGFP positive cells and pHes5-EGFP positive cells. (**A**) Schematic diagram of comparison of the gene expression. Venn diagram showing overlapping of genes of pHes1-EPG at P1 and P4 (upper panel), and these genes with pHes5-EGFP C1 genes^27^ (middle panel), and these genes with pHes5-EGFP C10 genes^27^ (lowest panel). (**B**) The genes enriched in Müller glia and/or in astrocytes^27^ are shown in the left column, and colors in the right column indicate which subgroup the genes were found. CRLBP-1 and Gnb1l were not listed in both pHes1-EGFP and pHes5-EGFP gene list.

A previous study examined Müller glia specific gene expression in pHes5-EGFP mouse retina^27^, and we asked whether genes identified in our study and their study overlapped or not. Microarray analysis was employed to obtain comprehensive gene expression pattern in pHes5 drove GFP positive retinal cells from P0, to P21, and hierarchical clustering was done^27^. Cluster 1 contained genes that were expressed highly in pHes5-EGFP retinas at all ages, and cluster 10 contained genes that tended to increase in expression over the first three postnatal weeks^27^. We compared the genes listed in clusters 1 and 10 in the previous study with our 952 and 1,077 genes that were expressed higher in pHes1_EPCs than in pHes1_ENCs (Fig. 2C). Interestingly, only a small percentage of the genes were in common between all four comparisons, but transcription factors were among the genes listed in common (Fig. 7A). However, we found that most of the genes enriched in Müller glia and/or in astrocytes^27^ were included in either the 952 or 1,077 gene categories (Fig. 7B).

## Discussion

In this report, we characterized the Müller glia lineage-specific molecular signature in the developing retina. Purifying retinal cells using *Hes1* promoter-dependent EGFP expression enriched the Müller glia, and we examined the genes expressed specifically in that fraction using RNA-seq. We initially defined Müller glia-specific genes by comparing the gene expression patterns between the pHes1-EGFP positive- and negative-fractions; however, the majority of pHes1-EGFP-specific genes were not upregulated after commitment to the Müller glia lineage, rather, these genes were downregulated in other cell lineages. These results suggest that progenitor cells and Müller glia share gene expression patterns. De-differentiation of Müller glia has been reported under various experimental conditions^28–30^, and a common gene expression pattern may support the ability of Müller glia to be reprogrammed to progenitor-like cells.

By combining a previously constructed ChIP-seq database of Cd73-positive and -negative developing retinal cells, we examined histone modification of gene loci that were enriched in pHes1-EPCs. The genes were subgrouped by their histone modification patterns, and interestingly, transcription factors were highly enriched in the clusters of genes in which their loci were highly H3K27me3 modified. Therefore, we speculate that H3K27 methylation regulates the expression of a particular set of transcription factors. Previously, we obtained results indicating an earlier timing for differentiation of Müller glia in retina-specific *Ezh2* knockout mice^3,7^; therefore, the role of H3K27me3 in defining the time when Müller glia differentiation is initiated was surmised. However, H3K27me3 appeared to act as a shut-off mechanism for critical transcription factors playing roles in Müller glia differentiation in other lineages. This phenomenon was clearly indicative of regulation of *Hes1* expression. In fact, modification of H3K27me3 at the *Hes1* locus increased significantly with retinal development, and *Ezh2* knockout released *Hes1* transcription from suppression. It is interesting that the level of H3K27me3 modification at the *Hes5* locus was very low at all developmental stages examined, although the time course of the *Hes5* expression pattern was quite similar to that of *Hes1*. The *Hes1* and *Hes5* genes are transactivated by the Notch intracellular domain complex with Rbpj and Maml^31^; therefore, mechanisms to distinguish *Hes1* and *Hes5* gene loci for modifying H3K27me3 may be other than Notch related molecules.

The comparison of genes expressed in pHes1-EGFP-positive cells and pHes5-EGFP-positive cells suggested differences in *pHes1* and *pHes5* labeled cells. GFP was expressed in the central retina in pHes1-Tg mice, but was absent from peripheral regions, and pHes5-Tg mice expressed GFP more strongly in the peripheral retina, beginning at E13.5^14^. The comparison of gene expression patterns in pHes1-EGFP-positive cells and pHes5-EGFP positive cells indicated that the major genes expressed in Müller glia were included in common in both lists; however, only a small fraction of all genes were listed in common. Because the methods used to analyze the genes (i.e., microarray vs. RNA-seq), the retinal developmental stage, and the bioinformatics methods used to list the Müller glia-specific genes differed between the two studies, currently we could not conclude that the difference of gene expression patterns was due to differences in the *Hes1*-positive and *Hes5*-positive cell populations. However, as the distributions of the two populations were quite different, we cannot exclude the possibility that peripheral and central Müller glia have different characteristics represented by different gene expression patterns.

The major H3K27 methyltransferase in the retina is *Ezh2*, but *Ezh1* is also expressed at a later developmental stage^3^. The H3K27me3 level at the *Hes1* locus increased in the Cd73_N fraction and maintained high level in Cd73_P fraction, but *Ezh2* expression decreases in Cd73_N and Cd73_P cells as postnatal retinal development proceeds^13^. *Ezh1* expression was weak but constant; however, shRNA based loss-of-function of *Ezh1* rather decreased *Hes1* level (Supplemental Fig. 4A,B), and *Ezh1* knockout mice produced comparable levels of the *Hes1* transcript in the adult retina (data not shown), suggesting that *Ezh1* is less likely to have a role sustaining the H3K27me3 level during late retinal development. We hypothesize involvement of other enzyme(s) in H3K27 methylation during late differentiation and to maintain the mature retina.

## Electronic supplementary material

Supplementary Information

Table S1

Table S2

Table S3

Table S4

Table S5
