# Author Correction: Analysis of Müller glia specific genes and their histone modification using Hes1-promoter driven EGFP expressing mouse

**DOI:** 10.1038/s41598-018-27041-9

**Published:** 2018-06-07

**Authors:** Kazuko Ueno, Toshiro Iwagawa, Genki Ochiai, Hideto Koso, Hiromitsu Nakauchi, Masao Nagasaki, Yutaka Suzuki, Sumiko Watanabe

**Affiliations:** 1 Division of Molecular and Developmental Biology, Institute of Medical Science, University of Tokyo, Tokyo, Japan; 2 Division of Biomedical Information Analysis, Department of Integrative Genomics, Tohoku Medical Megabank Organization, Tohoku University, Sendai, Miyagi Japan; 3 Division of Stem Cell Therapy, Center for Stem Cell Biology and Regenerative Medicine, Institute of Medical Science, University of Tokyo, Tokyo, Japan; 4 Department of Medical Genome Sciences, Graduate School of Frontier Sciences, University of Tokyo, Chiba, Japan

Correction to: *Scientific Reports* 10.1038/s41598-017-03874-8, published online 15 June 2017

Five supplementary datasets containing gene sequencing data were omitted from the original version of this Article. This has been corrected in the HTML version of the Article; the PDF version was correct at time of publication.

In addition, in the Results section, under the subheading ‘Comprehensive analysis of the gene expression patterns of Hes1 promoter (pHes1)-driven EGFP-positive and -negative populations’,

“To verify the RNA-seq data, we performed unsupervised K-means clustering and all genes in the RNA-seq to generate four clusters, which provided good resolution (Table S1, since excel files are not allowed to upload, I deposit all supplemental tables in the following drive; https://drive.google.com/drive/folders/0B1G_egGb4spiZmZJNWhYemRvRFk?usp=sharing).”

now reads:

“To verify the RNA-seq data, we performed unsupervised K-means clustering and all genes in the RNA-seq to generate four clusters, which provided good resolution (Table S1).”

Additionally, in the Results section, under the subheading ‘Hes1, but not Hes5, is suppressed by modifying H3K27me3 during the late phase of retinal development’,

“On the other hand, in Cd73_NPs, H3K27me3 level was upregulated during retinal development to actively suppress *Hes1* expression.”

now reads:

“On the other hand, in Cd73_NCs, H3K27me3 level was upregulated during retinal development to actively suppress *Hes1* expression.”

These errors have now been corrected in the PDF and HTML versions of the Article.

