# Supplementary material for: Analysis of Müller glia specific genes and their histone modification using Hes1-promoter driven EGFP expressing mouse
Source: Sci Rep. 2017 Jun 15;7:3578. doi: 10.1038/s41598-017-03874-8 (PMC5472600; doi:10.1038/s41598-017-03874-8)

***Supplementary Information***

***Analysis of Müller glia specific genes and their histone modification using Hes1-promoter driven EGFP expressing mouse***

Authors: Kazuko Ueno*1, 2, Toshiro Iwagawa*1, Genki Ochiai1, Hideto Koso1, Hiromitsu Nakauchi3, Masao Nagasaki2, Yutaka Suzuki4, and Sumiko Watanabe1

**Supplemental Fig. 1**

**Schematic diagram of the flow of analyses in this work and definition of the cells and genes.**

**
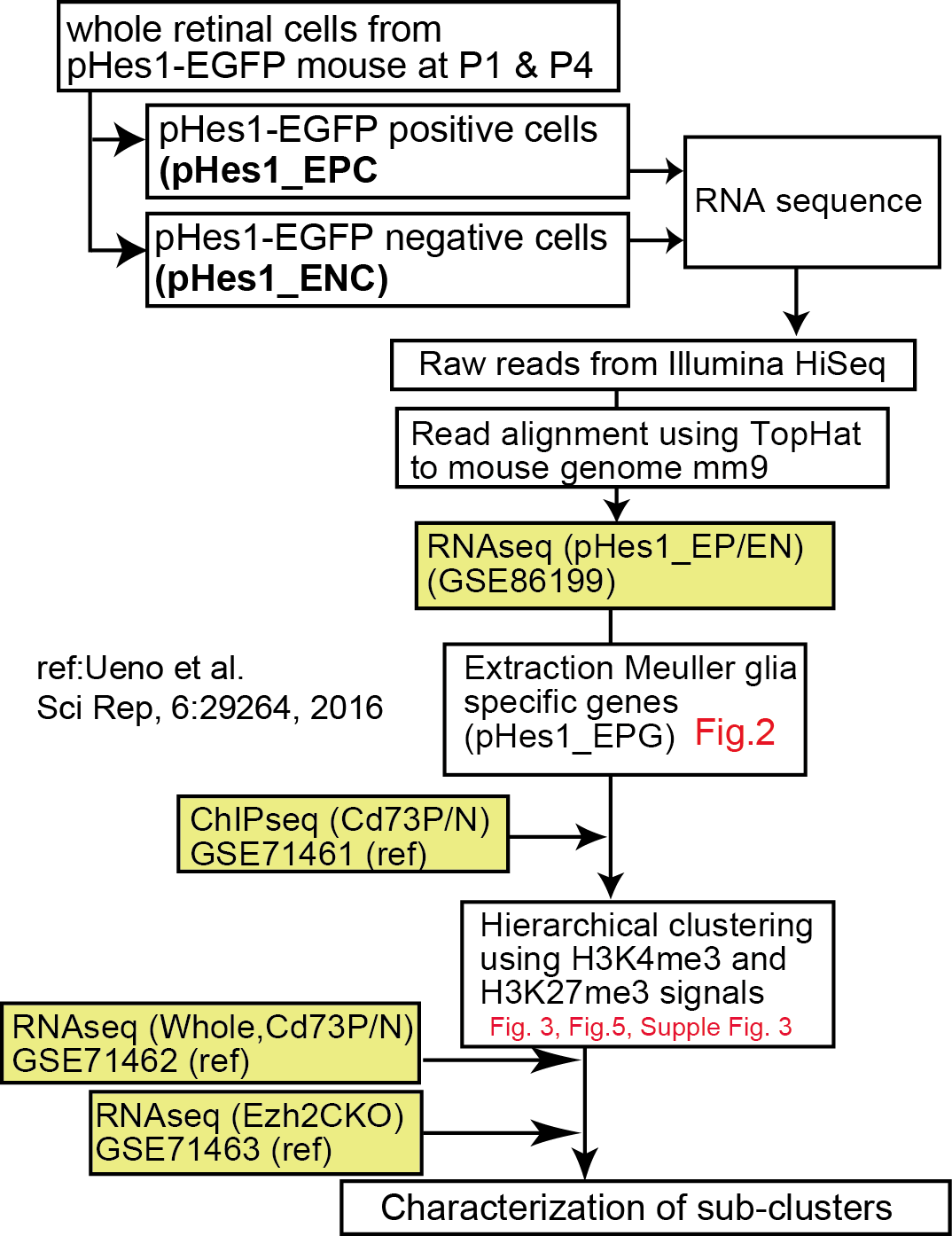
**

**Supplemental Fig. 2 Immunostaining patterns of frozen sectioned retina of *pHes1*-dEGFP mouse at 8 weeks. Antibodies for EGFP, and Müller glia markers, GS (A) and Ccnd3 (B) were used. Scale bar = 50 μm.**


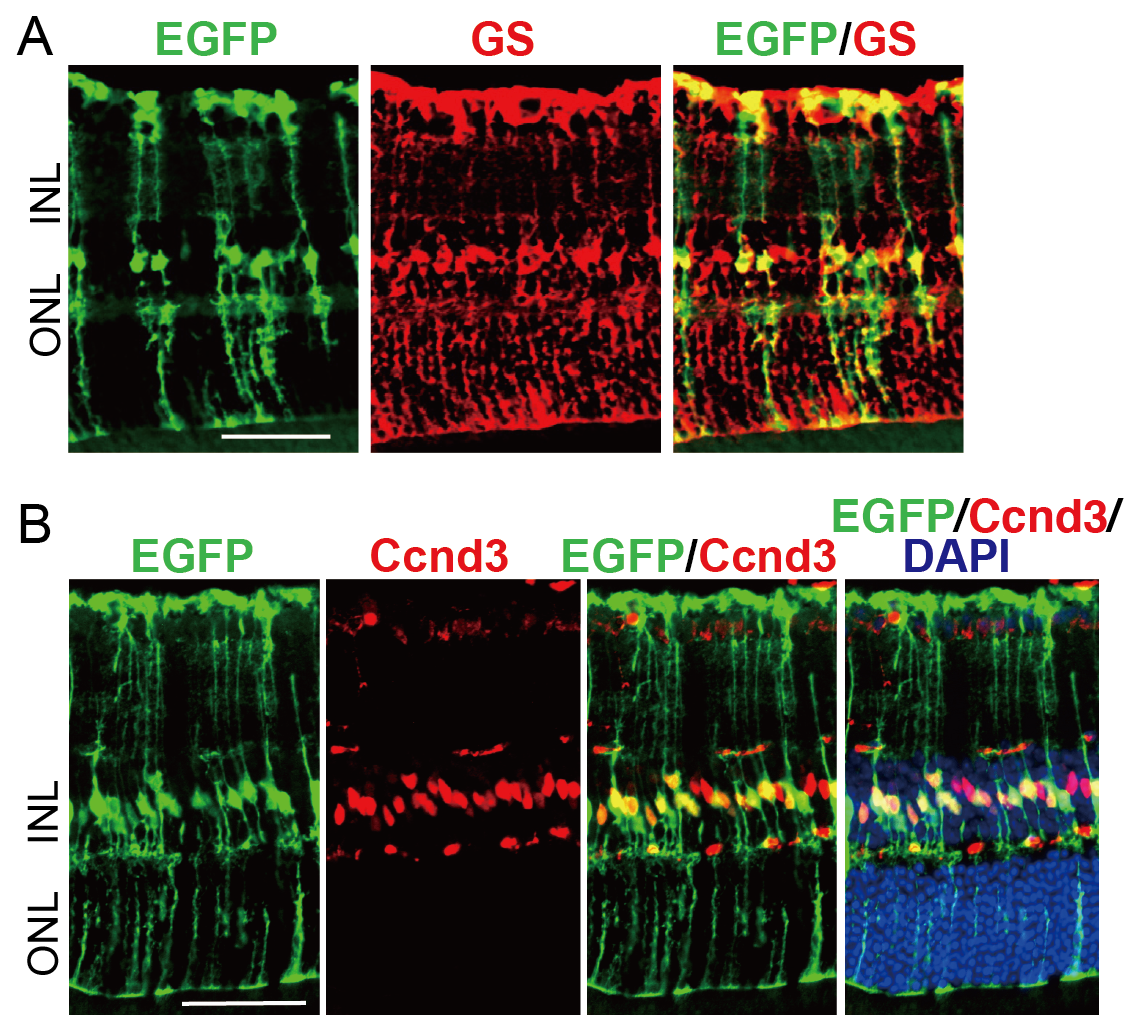


**Supplemental Fig. 3 Characterization of genes upregulated in pHes1 EGFP positive cells in P1 and P4. A-C. Distribution of H3K4me3 (ChIP-seq, A), H3K27me3 (ChIP-seq, B), and transcripts (RNA-seq, C) levels of genes in each sub-cluster. ChIP-seq and RNA-seq were performed by using Cd73P and Cd73N retinal sub-fractions at P2, P5, and P8. D. Percentage of genes categorized to transcription factors by IPA analysis in total number of genes in the clusters.**


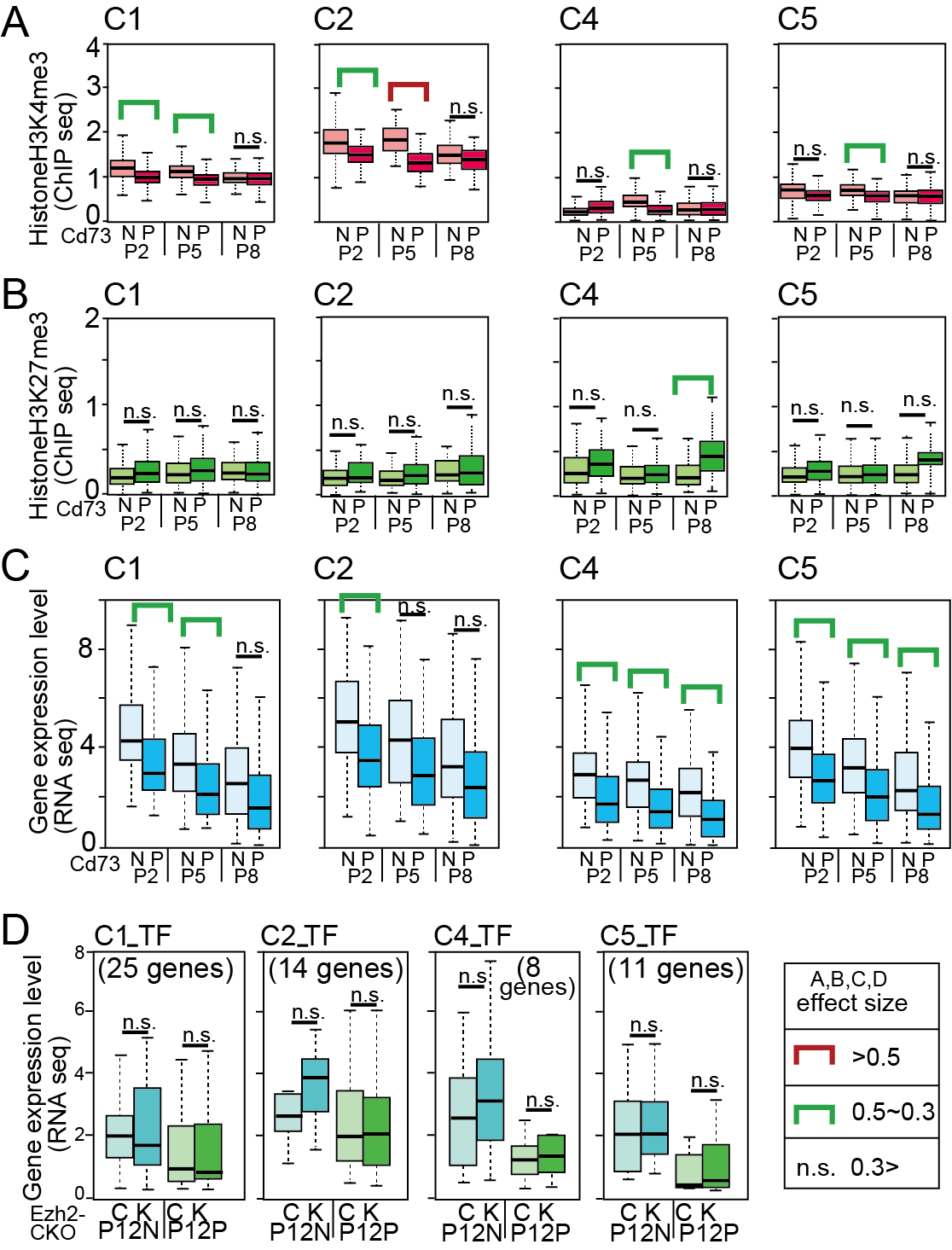


**Supplemental Fig. 4 A. Plasmids encoding sh-RNA against *Ezh1* (sh-Ezh1_1, sh-Ezh2_2), were transfected into NIH3T3 cells and after 48 hours, cells were harvested, and expression of *Ezh1* and *Ezh2* were examined by RT-qPCR. B. The plasmids were transfected into retinal explants prepared from mouse embryos at E18. After 9 days of culture, the explants were harvested, and mRNA levels of indicated genes were examined by RT-qPCR.**


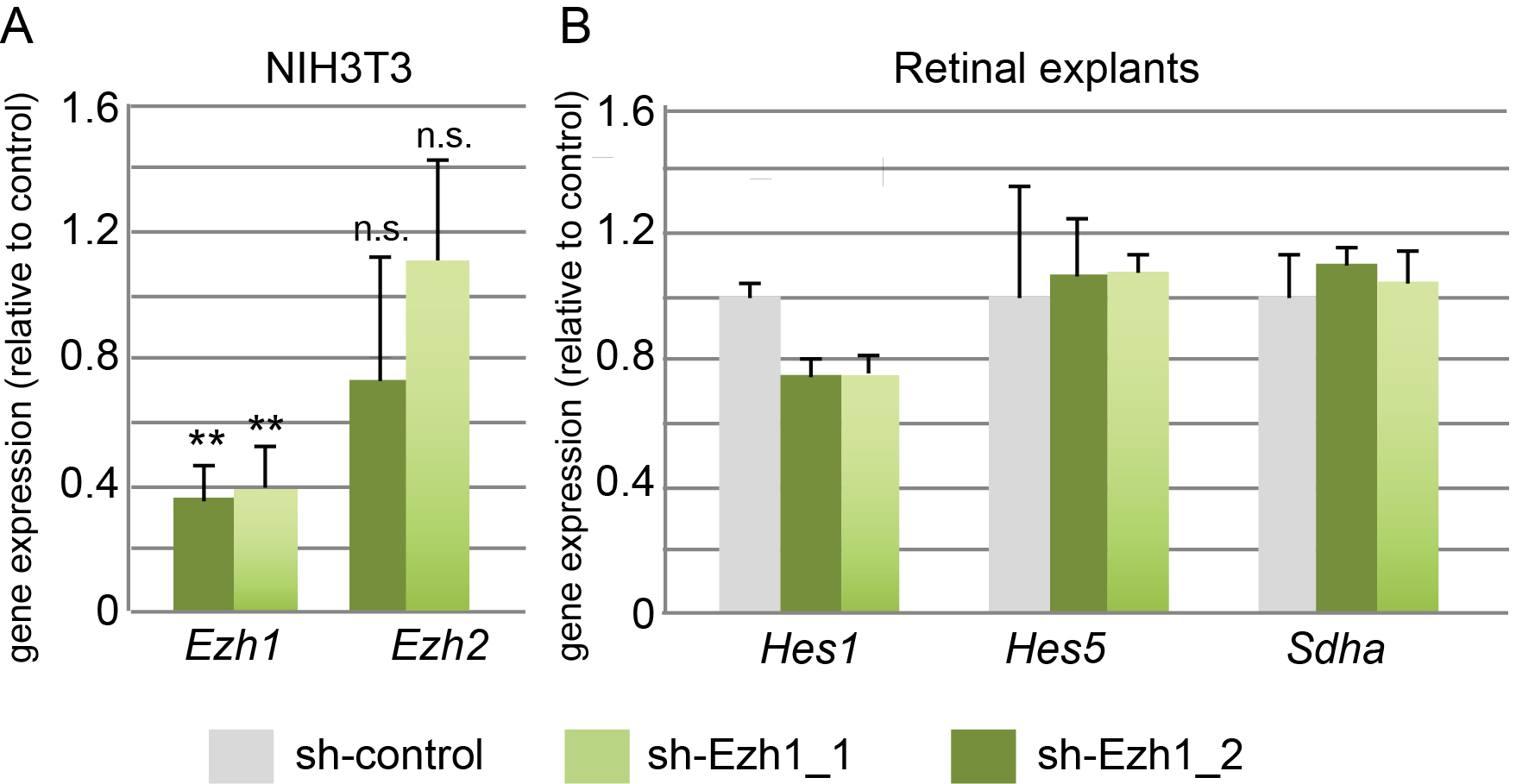

Supplement: Supplementary file 1 — Supplementary Information [file 41598_2017_3874_MOESM1_ESM.doc]
